# Effects of cTBS on the Frequency-Following Response and Other Auditory Evoked Potentials

**DOI:** 10.3389/fnhum.2020.00250

**Published:** 2020-07-08

**Authors:** Fran López-Caballero, Pablo Martin-Trias, Teresa Ribas-Prats, Natàlia Gorina-Careta, David Bartrés-Faz, Carles Escera

**Affiliations:** ^1^Institute of Neurosciences, University of Barcelona, Barcelona, Spain; ^2^Cognitive Neuroscience Research Group, Department of Clinical Psychology and Psychobiology, University of Barcelona, Barcelona, Spain; ^3^Medical Psychology Unit, Department of Medicine, Faculty of Medicine and Health Sciences, University of Barcelona, Barcelona, Spain; ^4^Institut de Recerca Sant Joan de Déu (IRSJD), Barcelona, Spain; ^5^Institut d’Investigacions Biomèdiques August Pi i Sunyer (IDIBAPS), Barcelona, Spain

**Keywords:** frequency-following response, neural generators, auditory cortex, transcranial magnetic stimulation, continuous theta burst stimulation

## Abstract

The frequency-following response (FFR) is an auditory evoked potential (AEP) that follows the periodic characteristics of a sound. Despite being a widely studied biosignal in auditory neuroscience, the neural underpinnings of the FFR are still unclear. Traditionally, FFR was associated with subcortical activity, but recent evidence suggested cortical contributions which may be dependent on the stimulus frequency. We combined electroencephalography (EEG) with an inhibitory transcranial magnetic stimulation protocol, the continuous theta burst stimulation (cTBS), to disentangle the cortical contribution to the FFR elicited to stimuli of high and low frequency. We recorded FFR to the syllable /ba/ at two fundamental frequencies (Low: 113 Hz; High: 317 Hz) in healthy participants. FFR, cortical potentials, and auditory brainstem response (ABR) were recorded before and after real and sham cTBS in the right primary auditory cortex. Results showed that cTBS did not produce a significant change in the FFR recorded, in any of the frequencies. No effect was observed in the ABR and cortical potentials, despite the latter known contributions from the auditory cortex. Possible reasons behind the negative results include compensatory mechanisms from the non-targeted areas, intraindividual variability of the cTBS effectiveness, and the particular location of our target area, the primary auditory cortex.

## Introduction

The frequency-following response (FFR) is a sustained evoked potential recorded with electroencephalography (EEG) or magnetoencephalography (MEG) that mimics the periodic features of the auditory stimulus waveform. It appears after the transient V—a complex of the phasic auditory brainstem response (ABR), for which it is sometimes described as the sustained part of the ABR (Skoe and Kraus, 2010). FFR is thought to reflect phase-locked neural activity of the auditory system to the spectral and temporal components of the acoustic signal (Krishnan et al., 2004; Chandrasekaran and Kraus, 2010; Skoe and Kraus, 2010) and can be elicited by different types of stimuli, such as pure tones, vowels, and syllables. Moreover, it is sensitive to both the fine structure and the envelope of the signal. Given the properties of the FFR, it has been widely studied in the field of auditory neuroscience and is considered a useful non-invasive tool to explore the neural mechanisms behind the representation of incoming sounds in the hearing brain.

FFR has been shown to be sensitive to different phenomena related to auditory perception and, in turn, to higher-level processing of language and music. This includes speech-in-noise perception (Du et al., 2011; Chandrasekaran et al., 2014), pitch discrimination training (Carcagno and Plack, 2011), rapid auditory learning (Skoe et al., 2013), and language experience and bilingualism (Krishnan et al., 2005; Kraus and Chandrasekaran, 2010; Krizman et al., 2012), musical training (Parbery-Clark et al., 2011; Skoe and Kraus, 2012; Bidelman, 2013; Bidelman and Alain, 2015), as well as age-related changes in auditory abilities (Anderson et al., 2013; Bidelman et al., 2014). Moreover, FFR is sensitive to task-related attention (Hairston et al., 2013) and stimulus probability (Skoe et al., 2014), and it is modulated by processes of regularity encoding, temporal predictability (Gorina-Careta et al., 2016), and deviance detection (Slabu et al., 2012; Escera, 2017). On the other hand, FFR has been shown to be affected in several clinical conditions, such as hearing impairment (Bellier et al., 2015), language impairment (Rocha-Muniz et al., 2012), reading disorders (Chandrasekaran et al., 2009; Billiet and Bellis, 2011), autism (Otto-Meyer et al., 2018; Font-Alaminos et al., 2020), and mild cognitive impairment (Bidelman et al., 2017). Furthermore, on the genetic aspects of the FFR, the involvement of the serotonin transporter expression has been revealed (Selinger et al., 2016).

To date, the neural generators of the FFR remain under debate. Yet, in order to properly interpret the results obtained by the studies mentioned above, it is critical to elucidate the contributions from different cerebral structures to the scalp-recorded signal. Traditionally, converging evidence from human and animal studies pointed to a subcortical origin of the FFR. Human EEG studies have shown that a high number of averages are needed to obtain a reliable response, suggesting a low signal-to-noise ratio (SNR) of the signal, and human lesion studies showed that no FFR could be obtained from patients with upper brainstem lesions (Sohmer et al., 1977). Additionally, evidence from source-reconstruction techniques with EEG revealed major contributions to the FFR from the midbrain (Bidelman, 2015). In line with the research conducted with humans, animal studies using single-unit recordings have shown that very early in the auditory processing, structures of the auditory pathway represent the incoming stimuli with high precision (Langner, 1992; Nelken, 2004), resembling FFR characteristics. Moreover, first-spike latencies in the inferior colliculus (IC) of cats align with the onset latency of the FFR (Schreiner and Langner, 1988), with a phase correspondence between FFR and single-unit activity in the cochlear nucleus and superior olivary complex (Marsh et al., 1974). Still in cats, cryogenic cooling of the IC was shown to reduce the FFR (Smith et al., 1975). Some authors concluded that, in that species, ∼95% of the scalp-recorded FFR can be attributed to activity from the cochlea, the cochlear nuclei, and the superior olivary nuclei (Gardi et al., 1979). Furthermore, findings from several studies performing intracranial recordings in awake monkeys suggested that the upper phase-locking limit in cortical neurons could be of ∼100 Hz in these species (Steinschneider et al., 1980, 2007), below the phase-locked activity recordable with FFR (e.g., Aiken and Picton, 2008), thus contributing to the notion that FFR would not have a cortical origin.

Despite all of the evidence pointing to a subcortical origin of the FFR, in a recent human study using MEG a strong cortical contribution was found for FFRs recorded to speech syllables of 98 Hz fundamental frequency (F0), especially in the right hemisphere (Coffey et al., 2016). Similar findings were obtained in an even more recent MEG study (Hartmann and Weisz, 2019), in which, in addition, cortical contributions to the FFR from the right hemisphere were the only ones modulated by intermodal attention. Still with MEG data, in Ross et al. (2020), source analysis revealed a faithful phase-locked representation of the speech stimulus’ F0 (100–140 Hz range) in auditory cortices, although with further analysis combining EEG and MEG and comparing F0 and N1 responses, authors estimated that approximately one-third of the scalp-recorded F0 would be of cortical origin. Overall, such results would help reinterpret the already mentioned findings of FFR modulation by factors theoretically associated with cortical plasticity, such as musical training or bilingualism. However, a crucial aspect arises when interpreting results in FFR studies, and that is the frequency of stimulation used. Notably, phase-locking capacities of neurons along the auditory pathway are progressively reduced from brainstem to cortical levels, with a suggested ∼100-Hz limit at the cortex (Joris et al., 2004). Theoretically, this would imply that FFR sources vary depending on the frequency of the stimulus and that FFR recorded to stimuli with frequencies above 100 Hz should be free of cortical contributions. In this regard, a recent study using source-reconstruction techniques with EEG (Bidelman, 2018) found that FFR contributions from the primary auditory cortex (PAC) were present for the stimulus’ fundamental frequencies up to 150 Hz but disappeared for harmonics above that limit, for which only bilateral auditory nerve and IC contributions remained. Incidentally, these frequency cutoffs must be taken with caution, as conclusive evidence in humans has not yet been established. Studies performing intracranial recordings of the auditory cortex in epilepsy patients found phase-locking activity to speech stimuli of frequencies up to 120 Hz (Behroozmand et al., 2016) and to click trains of 200 Hz (Brugge et al., 2009; Nourski et al., 2013). Moreover, the mechanism described by the volley principle theory (Wever and Bray, 1937), would allow the encoding of high frequencies in cortical neurons.

Importantly, both EEG and MEG spatial resolution is low, since the signal recorded at the sensor level is the result of overlapping brain signals from different anatomical sites, and source-reconstruction techniques have limitations, as they require to solve an inverse problem with infinite possible solutions (Mahjoory et al., 2017). Given these limitations, in the present study we addressed the question of the anatomical sources of the FFR from a different perspective, trying to complement findings from inverse solution methods. Instead of reconstructing the sources from the scalp-recorded signal, we recorded FFR before and after a transient inactivation of the right primary auditory cortex, by means of the repetitive transcranial magnetic stimulation (rTMS)-patterned protocol known as continuous theta burst stimulation (cTBS; Huang et al., 2005). The cTBS protocol can modulate cortical excitability producing long-term depression-like phenomena, resulting in a downregulation of the cortical activation of the targeted region (e.g., Tupak et al., 2013). Using the measurable output of motor evoked potentials, a recent meta-analysis showed that the inhibitory post-effects of cTBS may remain significant after 30 min of stimulation, depending on the protocol employed (see review by Chung et al., 2016). Larger effect sizes are typically found during the first 5–10 min after cTBS administration, with inhibition linearly returning back to baseline (see review by Wischnewski and Schutter, 2015). In addition, neuronavigated rTMS has been successfully applied in a safe and precise manner to target primary (e.g., Schecklmann et al., 2016) and secondary (e.g., Slotema et al., 2014) auditory cortices. Moreover, cTBS targeting the right primary auditory cortex (Heschl’s gyrus) has been shown to modulate BOLD responses in the auditory cortex (Andoh and Zatorre, 2013), with measurable performance changes in tasks related with auditory processing (Andoh and Zatorre, 2011). Together, these results, along with the cortical contribution of the MEG-recorded FFR being more prominent in the right hemisphere (Coffey et al., 2016; Hartmann and Weisz, 2019; Ross et al., 2020), support the selection of the right primary auditory cortex as a target for cTBS in our study.

The goal of the present study was hence to disentangle whether the right primary auditory cortex contributes to the scalp-recorded FFR, as well as to test whether this potential contribution is dependent on the frequency of the stimulus used to elicit the FFR (Low, 113 Hz; or High, 317 Hz). Our theoretical prediction was that FFR elicited to the low frequency would be modulated by the transient inactivation of the right primary auditory cortex with cTBS, whereas FFR to the high frequency would remain unaffected. As control conditions in our design, we also assessed whether the transient inactivation of the right primary auditory cortex would affect the auditory brainstem response (ABR) and cortical potentials (P50, N1, and P2 components), to confirm whether cTBS in that area would induce changes in cortical evoked potentials, while not affecting subcortical ones (ABR).

## Materials and Methods

### Participants

Twenty participants (11 males), ranging in age from 18 to 34 years (mean = 24.3; standard deviation = 4.2), were included in the study, recruited among University of Barcelona students. All included participants, but one, were naïve to previous TMS administration and right handed (Edinburgh Handedness Inventory >40) to minimize variability in the localization of language areas (Knecht et al., 2000) and avoid a potential confound with our target area for cTBS. Exclusion criteria included history of neurologic or psychiatric condition, abnormal MRI structural measurements, and abnormal hearing thresholds. A pure-tone audiometry (frequency range: 250–4,000 Hz), using audiometric Beyerdynamic DT48-A headphones (Heilbronn, Germany), was performed for each participant at the screening session and before each experimental session, ensuring mean hearing thresholds below 20 dB NHL at each ear. In accordance with TMS safety guidelines (Rossi et al., 2009), pregnancy, previous history of losing consciousness, and prior experience of a seizure or diagnosis of epilepsy were also among the exclusion criteria. In addition, participants with more than 5 years of musical training in the last 5 years before the study were also discarded, as musical training is known to modulate the FFR (e.g., Skoe and Kraus, 2012). Furthermore, in screening sessions, five participants were discarded due to hardly detectable FFRs, two due to the presence of post-auricular muscle response (PAM) artifact and two decided not to participate in the study as they considered the cTBS pulse to be annoying. The experimental protocol was approved by the Bioethics Committee of the University of Barcelona and was in accordance with the WMA Declaration of Helsinki Ethical Principles for Medical Research Involving Human Subjects. At the beginning of the screening session, written informed consent was obtained from each participant after all the details of the research (except the hypotheses) were explained to them, including the characteristics of the EEG, MRI, and TMS methods and the possibility to withdraw from the experiment at their wish. Upon completion of the four sessions of the study, they were compensated by monetary payment with 80€.

### Procedure and Experimental Design

**FIGURE 1:**
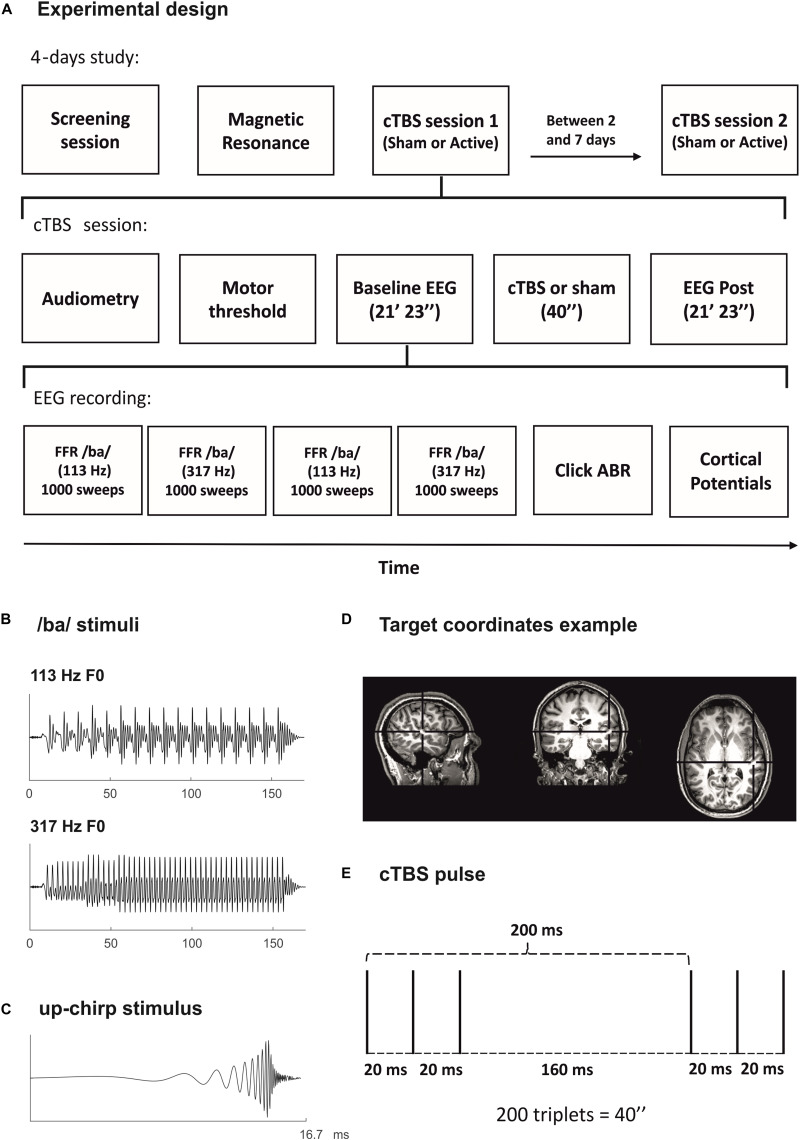
**(A)** Experimental design. **(B)** Stimulus waveform for the syllable /ba/ of low frequency (F0: 113 Hz; top) and of high frequency (F0: 317 Hz; bottom), used to elicit the FFR. **(C)** Stimulus waveform for the up-chirp (Dau et al., 2000), used to elicit the cortical potentials. **(D)** Representation of the continuous theta burst stimulation (cTBS) TMS protocol. Three pulses at 50 Hz (bursts) presented every 200 ms during 40″ (600 pulses in total). **(E)** Example of target coordinates for the cTBS pulse in a participant’s MRI. Right primary auditory cortex MNI coordinates (*x*-, *y*-, *z*-) of 50, –21, and 7, transformed into the participant’s native MRI space.

The study was conducted in four sessions for each participant, in separate days: screening session, MRI session, and two experimental sessions (Sham and Active). The rationale for using a session with sham TMS was to discard that the potential differences in our EEG measures before and after TMS could be attributed to factors such as the repetitive auditory stimulation or the noises produced by cTBS administration. The order of Sham and Active sessions was counterbalanced across participants, and they were separated by a minimum of 2 days and a maximum of 7 (study design represented in Figure 1A). During the screening session, after the audiometry, FFR and recordings of cortical potentials were obtained from each participant, ensuring that FFR to both low- and high-frequency stimuli could be detected as well as the absence of PAM response. Because of our EEG acquisition montage, PAM response could not be cleaned off-line in our data. Thus, it was crucial to identify participants displaying this kind of artifact beforehand. During the screening session also, we determined resting and active motor thresholds (rMTH and aMTH) for each participant, using a template MRI for neuronavigation, and applied a maximum of 4 s of the cTBS protocol placing the coil in the approximate position of the head where it would be placed in the experimental sessions (T4 electrode location according to the 10–20 EEG electrode system). With this, we aimed to allow participants to familiarize with the TMS before the real experiment and to let us know how much discomfort it produced due to the proximity of the coil to the ear and ocular nerves. During the MRI session, the structural MRI from each participant was acquired. Participants who were already in possession of their structural MRI did not participate in this session. Sham and Active experimental sessions were identical except for the coil with which the cTBS pulse was applied, either the real one or the sham. In these sessions, after the audiometry, rMTH and aMTH were determined for each participant. Then, Baseline and Post EEG recordings were performed and, in between the two, the cTBS pulse was applied at the target coordinates of stimulation for each participant. Neuronavigation in experimental sessions was performed using participants’ MRI. Both Baseline and Post EEG recordings consisted in four FFR blocks, two for each stimulation frequency, followed by the click ABR and the cortical potentials blocks. The starting frequency of the FFR recordings (low or high) was counterbalanced across participants. Instead of using two FFR blocks, one for each frequency, the reason to divide FFR recordings in four blocks, two for each frequency, was to avoid FFRs to a particular frequency to be more affected by the cTBS pulse, as inhibitory cTBS effects fade away linearly with time (Wischnewski and Schutter, 2015; Chung et al., 2016). During EEG recordings, participants were seated comfortably and instructed to perform a visual attention task while listening to the sounds, ensuring they were not paying attention to the auditory stimuli (minimum of 80% hit rate in the visual task). The task consisted in the random presentation of numbers from 2 to 9, with a SOA jittered between 850 and 1,100 ms. During the visual task, participants had to press the ENTER key as fast as possible only when the same number appeared twice in succession (20% times). They were instructed to tap gently to avoid myogenic artifacts. The visual task was concurrent with every EEG block, so the duration of the task was dependent on the duration of the EEG block. Participants were asked to refrain from alcohol intake and from taking any drugs during the 24 h before any of the four sessions of the study. All sessions but the MRI one were held at the premises of the Medical Psychology Unit, located in the Faculty of Medicine and Health Sciences of the University of Barcelona.

### Stimuli

For FFR recordings, the stimulus was the consonant-vowel (CV) syllable /ba/, created with the Klatt-based synthesizer (Klatt, 1976). The syllable duration was 170 ms, with a 10-ms onset period, 45-ms consonant transition, and 115-ms steady-state part, corresponding to the vowel. The fundamental frequency (F0) was modified with Praat 6.0.10 software (Boersma, 2002; Boersma and Weenink, 2016) to create syllables with F0 of 113 Hz (low frequency) or 317 Hz (high frequency) (Figure 1B). The choice of these stimuli’s F0 was motivated, in the case of the low frequency, on its typical use in FFR literature (∼100 Hz, e.g., Rocha-Muniz et al., 2012; Slabu et al., 2012; Chandrasekaran et al., 2014; Coffey et al., 2016; Bidelman et al., 2017), and, in the case of the high frequency, on the fact that it is above the observed phase-locking capabilities of cortical neurons in the literature (see Joris et al., 2004; Behroozmand et al., 2016). The specific choice of these F0 values was also performed on purpose to avoid contamination with harmonics of the 50-Hz electric line in Europe. During the consonant transition, first (F1) and second (F2) formants rise from 737 to 842 Hz and from 1,436 to 1,650 Hz, respectively. In the steady-state part, both formants remain constant. The third formant (F3) stays at 3,170 Hz along all syllable durations. Syllables were presented at 85 dB SPL with a stimulus-onset asynchrony (SOA) of 270 ms.

For ABR recordings, the stimulus was a 0.1-ms square wave click, following recommended standards (Tsuchida et al., 2016). The stimulus was included in the default sound database of SmartEP platform (Intelligent Hearing Systems, Miami, Fl, EEUU). Clicks were presented at 85 dB SPL with a SOA of 52 ms.

For the recording of cortical potentials, the stimulus was an up-chirp (Dau et al., 2000), with a length of 16.7 ms (Figure 1C). It was created using MATLAB software (The MathWorks, Inc., Natick, MA, United States) and the Psychophysics Toolbox extensions (Brainard and Vision, 1997; Kleiner et al., 2007) by summing rising harmonic series of cosine waveforms from 50 to 8,000 Hz (Elberling et al., 2007). Chirps were presented at 70 dB SPL with a SOA of 500 ms.

All stimuli were presented to both ears, with alternating polarities and using Etymotic shielded insert earphones of 300 Ω (Etymotic Research, Inc., Elk Grove Village, IL, United States).

### MRI Acquisition

The anatomical magnetic resonance imaging (MRI) session took place at the Department of Diagnostic Imaging of Sant Joan de Déu Hospital (Barcelona, Spain). 3D structural datasets were acquired (T1 sequences, 240 slices, slice thickness of 1 mm) using a 1.5-T MRI scanner (Ingenia, Philips Medical Systems, Netherlands). Six participants of the sample were already in possession of their structural MRI from either a clinical examination or a previous study and voluntarily provided it for the purposes of the study. Quality standards of all structural MRI were of sufficient quality for the purpose of TMS neuronavigation.

### Neuronavigated TMS Protocol

The TMS was delivered with an eight-shaped coil using MagPro X100 magnetic stimulator (MagVenture A| S, Denmark). In all experimental sessions, stimulation was neuronavigated with a stereotactic system (eXimia Navigated Brain Stimulation, Nexstim, Finland) using individual MRI acquisition. rMTH and aMTH were determined for each participant before cTBS was applied. To do this, single TMS pulses were applied in the area of the right M1 cortical region corresponding to the left first dorsal interosseous (FDI) muscle, while motor evoked potentials (MEPs) were monitored through a pair of Ag-AgCl surface electrodes in a belly tendon montage, using AcqKnowledge 4.2 software and BIOPAC MP150 system (Biopac Systems, Inc., Goleta, CA, United States). Single pulses were administered starting at intensities corresponding to 35% of stimulator output capacity and increased in steps of 5% until reaching rMTH and aMTH values (Rossini et al., 2015). rMTH was defined as the minimum stimulus intensity that elicited at least 5 out of 10 consecutive MEPs of at least 50 μv peak-to-peak amplitude, whereas aMTH was defined as the minimum stimulus intensity that elicited at least 5 out of 10 consecutive MEPs of at least 200 μv peak-to-peak amplitude during FDI soft contraction (approximately 20% of maximum muscle contraction).

Continuous theta burst stimulation protocol consisted in the repeated application of triplets of pulses (bursts) at 50 Hz, with an inter-train interval (ITI) of 200 ms (5 Hz; theta), during 40″ (200 triplets, 600 pulses in total, Figure 1D), and its administration intensity corresponds to 80% of aMTH (Huang et al., 2005). This protocol has been described to produce a long-lasting (approximately 30 min) reduction in cortical excitability (Chung et al., 2016) and has been previously used to target primary auditory cortical areas (Schecklmann et al., 2016). The target location for the cTBS pulse was the right primary auditory cortex, Montreal Neurological Institute (MNI) coordinates (*x*-, *y*-, *z*-) of 50, −21, and 7. The coordinates of the stimulation target were defined individually by transforming them into the participant’s native MRI space, using the MNI template-to-native transformation matrix with FSL software (Figure 1E). In two participants’ MRIs, the transformation matrix was not properly implemented, as checked by visual inspection, and thus the target location was defined manually conforming to MRI-determined landmarks for Heschl’s gyri (Abdul-Kareem and Sluming, 2008). The coil was held tangentially to the skull, with the coil handle positioned upward, as described in previous studies targeting the auditory cortex with TMS (e.g., Schecklmann et al., 2016). For sham stimulation, a sham coil was used, mimicking the clicking sound of each TMS pulse. All TMS procedures were performed following international safety recommendations (Rossi et al., 2009), including cTBS only delivered in one single cerebral hemisphere and the use of earplugs during cTBS.

### EEG Acquisition

Electroencephalography recordings were performed using the SmartEP platform with cABR and Advanced Hearing Research modules (Intelligent Hearing Systems, Miami, F1, EEUU). Disposable snap Ag/AgCl electrodes were used, with one active electrode located at Cz according to the 10–20 EEG electrode system, a reference electrode placed at the left earlobe, and a ground electrode at the forehead. In four participants, the reference electrode was placed at the left mastoid instead, but the protocol was later changed to use the left earlobe due to the reduced probability of obtaining the PAM artifact with this reference. Nevertheless, none of these four participants had the PAM artifact and their EEG recordings were comparable to the rest of the sample. During the recordings, a tubular elastic net was placed on the participants’ head to help the fixation of the Cz electrode. All impedances were kept below 5 kΩ.

The duration of the stimulation blocks was automatically adjusted until the total number of intended artifact-free sweeps was obtained per block and participant. Overall, the number of rejected artifacts per block and participant was below 10%. In all EEG recordings, data was acquired with alternating polarities which were then averaged together (Aiken and Picton, 2008).

For FFR recordings, 4,000 artifact-free sweeps (in four blocks of 1,000 sweeps each) were acquired, 2,000 sweeps for each stimulation frequency (Low and High), with a sampling rate of 13,333 Hz. The total acquisition time was 4′ 30″ for each of the FFR blocks. Data was online bandpass filtered from 70 to 1,500 Hz, and the amplitude rejection criteria was ±30 μV. Data was epoched in time windows from −40.88 to 229.35 ms (baseline corrected).

For ABR recordings, 2,000 artifact-free sweeps were acquired, with a sampling rate of 40,000 Hz. Total acquisition time was 1′ 44″. Data was online bandpass filtered from 100 to 3,000 Hz, and the amplitude rejection criterion was ±30 μV. Data was epoched in time windows from −10.9 to 40.9 (baseline corrected).

For the recording of cortical potentials, 200 artifact-free sweeps were acquired, with a sampling rate of 6,666 Hz. Total acquisition time was 1′ 40″. Data was online bandpass filtered from 1 to 30 Hz, and the amplitude rejection criterion was ±80 μV. Data was epoched in time windows from −100.88 to 399 ms (baseline corrected).

### EEG Analysis

Data from the Cz electrode was analyzed using MATLAB software (The MathWorks, Inc., Natick, MA, United States). Average waveforms for FFR, ABR, and cortical potentials were obtained per participant, session (Sham or Active), measurement (Baseline or Post cTBS), and, in the case of FFR, frequency (low or high). FFRs obtained from the two FFR blocks of the same frequency (1,000 sweeps each) were averaged into a single FFR, leaving a single FFR for each stimulus frequency.

For the FFRs, different measures from both the time domain and the frequency domain were obtained, separately for each stimulus frequency, trying to portrait different aspects of this response as described in a recent study from our laboratory (Ribas-Prats et al., 2019). In that study, a detailed description on the aspects of the signal that each of these measures describe, as well as the way they were calculated, can be found. In the time domain, first, the stimulus-to-response cross-correlation (Pearson’s *r*) was calculated (Russo et al., 2004), yielding the magnitude of the first maximum cross-correlation value and its associated stimulus-to-response delay (neural lag). Second, the signal-to-noise ratio (SNR; Liu et al., 2015) with root-mean-square amplitude (μV) was calculated in three different portions of the FFR corresponding to the consonant transition (10–55 ms) and vowel (55–170 ms) regions of the syllable /ba/, as well as to the whole stimulus (0–170 ms), considering a baseline from −40 to 0 ms. To calculate SNR, consonant transition, vowel, and whole stimulus portions of the FFR were defined individually for every participant, accounting for the neural lag obtained from the stimulus-to-response cross-correlation. The range of neural lags obtained from all participants and conditions was 3 to 12.9 ms (mean = 8 ms; standard deviation = 2.03 ms). Third, a sliding time-window autocorrelation was computed, from which pitch error (Hz) and pitch strength (Pearson’s *r*) measures were extracted. To analyze FFRs in the frequency domain, a fast-Fourier transform (FFT), Hanning windowed, was computed over the three time windows previously defined (consonant transition, vowel, and whole stimulus), again adjusted, accounting for the individual neural lag. From the resulting spectra, first, amplitude values (μV/Hz) within a window of 10 Hz surrounding the F0 of the stimulus were retrieved (e.g., 108–118 for Low-frequency stimulus). Second, SNRs were calculated by dividing the mean amplitude over the 10-Hz window at F0 peak by the noise on the peak flanks. This noise was calculated as the mean amplitude of two 10-Hz windows at each side of the peak, separated by 20 Hz from the peak frequency window. All FFR analyses were performed with scripts developed in our laboratory based on analysis routines provided by Intelligent Hearing Systems (Miami, Fl, EEUU).

For the ABRs, mean amplitude values (μV) of wave V were retrieved, defining a time window from 5 to 6.5 ms from sound onset. For the cortical potentials, amplitude analyses were performed over three different components, P50 (30–50 ms), N1 (70–110 ms), and P2 (120–160 ms). For the analyses of both ABR and cortical potentials, time windows were defined based on peak values of the analyzed components on the grand-average waveforms.

### Statistical Analysis

Repeated-measures ANOVAs were performed separately for each of the FFR measures, the ABR wave V mean amplitudes, and the P50, N1, and P2 mean amplitudes. For FFR measures, a three-way repeated-measures ANOVA was performed, with the three levels being Session (Sham, Active), Measurement (Baseline, Post), and Frequency (Low, High). For ABR and cortical potentials, a two-way repeated-measures ANOVA was performed instead, with the two levels being Session and Measurement. For each of these comparisons, effect sizes were obtained using partial eta-squared, and whenever the assumption of sphericity was violated, degrees of freedom were corrected using Greenhouse–Geisser estimates.

Given our hypothesis, with these statistical comparisons, first, we expected a triple interaction between Session, Measurement, and Frequency levels in the FFR, with statistically significant differences between Baseline and Post measurements only occurring in the Active session and for the Low-frequency condition. Second, we did not expect interactions between Session and Measurement levels in the ABR, thus confirming no effect of cTBS at the brainstem level. Third, we expected an interaction between these two levels in the cortical potentials, with these cortical components only differing between Baseline and Post measurements in the Active session.

Additional statistical comparisons were computed in FFR, ABR, and cortical potentials. Specifically, we computed effect sizes between Baseline and Post measurements, separately for Active and Sham sessions and, in the case of FFR, separately for each frequency of stimulation as well. We did so by using Cohen’s *d*_rm_ as suggested by Lakens (2013). The formula used was as follows:

$$C\,o\,h\,e\,n^{\prime }\,s\,d_{\text{rm}}=\frac{M_{\text{diff}}}{\sqrt{S\,D_{1}^{2}+S\,D_{2}^{2}-2\times r\times S\,D_{1}\times S\,D_{2}}}\times \sqrt{2\times \left(1-r\right)}$$

*M*_diff_ is the difference between the mean (*M*) of the difference scores and the comparison value m (e.g., 0) and *r* is the correlation between measures. Confidence intervals (CI) for each effect size are reported. The CI provides information about the precision of an estimate and its potential generalizability or replicability (Banjanovic and Osborne, 2016). We used the bias-corrected and accelerated bootstrap (BCa) method with the Matlab function bootci (Efron and Tibshirani, 1993; DiCiccio and Efron, 1996). First, the effect size is computed in each of the 10,000 replications of the original sample. Next, the resulting bootstrap distribution is corrected for bias (i.e., skew) and acceleration (i.e., non-constant variance). Finally, the lower and upper bounds of the CI are found at the 0.025 and 0.975 quantiles of the corrected distribution.

## Results

### Frequency-Following Response (FFR)

**FIGURE 2:**
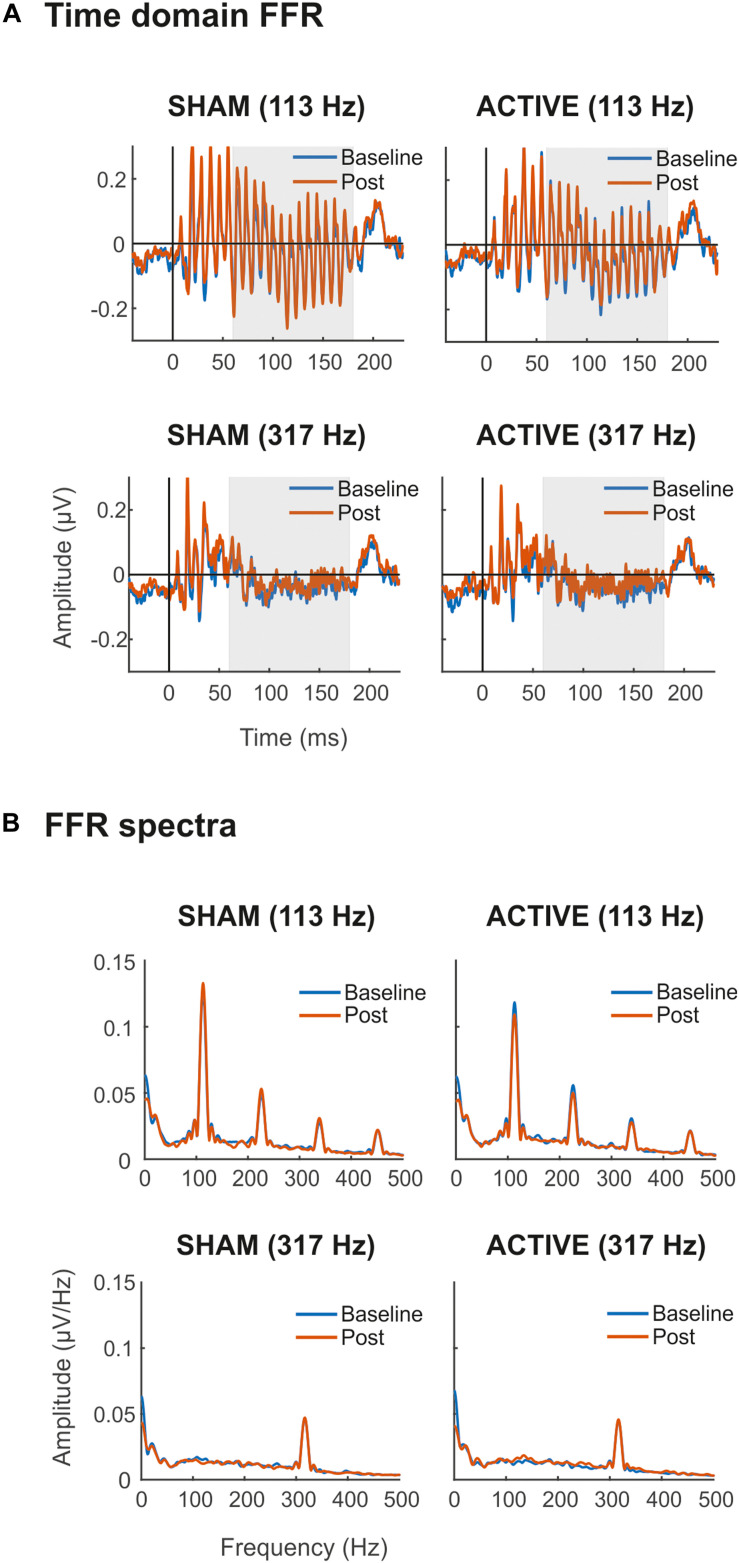
Time domain FFRs (μV) **(A)** and FFR spectra (μV/Hz) **(B)** elicited to syllable /ba/ with Low (113 Hz, top) and High (317 Hz, bottom) F0, in Sham (left side) and Active (right side) sessions. In blue, baseline FFR recordings before the cTBS pulse. In orange, Post cTBS FFR recordings. Shaded areas (65–180 ms) in time-domain FFRs represent time windows of the response corresponding to the vowel region of the stimulus, assuming 10 ms of neural lag, from where spectra were calculated for illustrative purposes. All recordings were obtained at the Cz electrode.

Grand-average FFRs are shown in Figure 2A. FFRs to both low- and high-frequency stimuli were obtained, with a clear spectral peak at stimulus F0 (Figure 2B). For the low-frequency stimulus, harmonics of F0 can also be observed. For illustrative purposes, spectra shown were calculated over the FFR portion corresponding to the vowel part of the stimuli (65–180 ms, assuming 10 ms of neural lag; shaded area in plots from Figure 2A).

Statistical comparisons of the time domain SNR revealed a main effect of the frequency factor in all three portions of the FFR [Transient, *F*_(__1_,_19__)_ = 69.53, *p* < 0.001, $\eta_{\text{p}}^{2}$ = 0.785; Constant, *F*_(__1_,_19__)_ = 60.27, *p* < 0.001, $\eta_{\text{p}}^{2}$ = 0.760; Total, *F*_(__1_,_19__)_ = 72.23, *p* < 0.001, $\eta_{\text{p}}^{2}$ = 0.792], indicating that the magnitude of the neural activity relative to the baseline was larger for FFR elicited to low-frequency stimuli. Moreover, overall differences in time domain SNR were found also between Baseline and Post EEG measurements in the consonant transition portion of the FFR (Table 1), indicating that the magnitude of the neural activity relative to the baseline changed across measurements. However, such difference was independent of the Session and Frequency factors, and therefore it could not be attributed to the cTBS pulse. Such differences were also found for the total portion of the FFR, but these did not survive for multiple-comparison correction. For the mean spectral amplitude at the FFR F0, again, statistical comparisons revealed a main effect of Frequency in all three portions of the FFR [Transient, *F*_(__1_,_19__)_ = 37.96, *p* < 0.001, $\eta_{\text{p}}^{2}$ = 0.666; Constant, *F*_(__1_,_19__)_ = 23.38, *p* < 0.001, $\eta_{\text{p}}^{2}$ = 0.552; Total, *F*_(__1_,_19__)_ = 35.45, *p* < 0.001, $\eta_{\text{p}}^{2}$ = 0.651], with higher spectral amplitudes in FFR to the low-frequency stimulus. Such effect was not found, as expected, when computing the spectral SNR [Transient, *F*_(__1_,_19__)_ = 1.080, *p* = 0.312, $\eta_{\text{p}}^{2}$ = 0.054; Constant, *F*_(__1_,_19__)_ = 0.871, *p* = 0.363, $\eta_{\text{p}}^{2}$ = 0.044; Total, *F*_(__1_,_19__)_ = 0.669, *p* = 0.424, $\eta_{\text{p}}^{2}$ = 0.034]. No significant effects in Session or Measurement factors, or in their interaction, were found for F0 mean spectral amplitude values or spectral SNR, as shown in Table 1, thus suggesting no effect of the cTBS pulse in these FFR measures.

**TABLE 1:** Results of repeated-measures ANOVA on FFR measures: time-domain SNR with root-mean-square amplitudes (SNR time domain), mean spectral amplitude of peak at F0 (F0 amplitude; μV/Hz), and SNR comparing the spectral peak at F0 with its flanks (SNR spectral F0).

FFR statistics	Transient			Constant			Total		
	*F*	*p*	$\eta_{\text{p}}^{2}$	*F*	*p*	$\eta_{\text{p}}^{2}$	*F*	*p*	$\eta_{\text{p}}^{2}$
**SNR time domain**									
Session	1.649	0.215	0.080	0.341	0.566	0.018	0.000	0.988	0.000
Measurement	19.16	**<0.001**	0.502	0.881	0.360	0.044	4.688	**0.043**	0.198
Ses*Meas	3.636	0.072	0.161	0.127	0.725	0.007	0.989	0.332	0.049
Ses*Meas*Fre	0.234	0.634	0.012	0.610	0.444	0.031	0.394	0.538	0.020
**F0 amplitude**									
Session	0.400	0.535	0.021	0.464	0.504	0.024	0.155	0.698	0.008
Measurement	0.659	0.427	0.034	0.007	0.936	0.000	0.020	0.889	0.001
Ses*Meas	0.566	0.461	0.029	0.219	0.645	0.011	0.538	0.472	0.028
Ses*Meas*Fre	2.005	0.173	0.095	0.185	0.672	0.010	0.496	0.490	0.025
**SNR spectral F0**									
Session	0.020	0.890	0.001	0.008	0.929	0.001	0.032	0.860	0.002
Measurement	1.194	0.288	0.059	0.343	0.565	0.018	0.180	0.676	0.009
Ses*Meas	0.286	0.599	0.015	1.727	0.204	0.083	0.459	0.506	0.024
Ses*Meas*Fre	2.149	0.159	0.102	1.985	0.175	0.095	2.392	0.138	0.112

Each measure was calculated at three portions of the FFR corresponding to different regions of the stimulus: consonant transition (Transient, 10–55 ms), vowel (Constant, 55–170 ms), and whole stimulus (Total, 0–170 ms), adjusted for each participant accounting for the individual neural lag. Session factor includes Sham and Active levels, Measurement factor includes Baseline and Post levels, and Frequency factor includes Low and High frequency levels. Session and Measurement factors, the interaction between them (Ses*Meas), and a triple interaction between Session, Measurement, and Frequency (Ses*Meas*Fre) are reported. For each factor and their interaction, F- and p-values are presented (degrees of freedom: 1,19) along with effect sizes ($\eta_{p}^{2}$). P-values below 0.05 are highlighted. p-values in the table are non-corrected for multiple comparisons.

To test more precisely whether the lack of effects in FFR measures would be expected in the population, comparisons between Baseline and Post values in these measures were performed by computing Cohen’s *d* and confidence intervals (CI) associated. Such analyses confirmed no effects in the main comparisons of interest given our hypothesis (i.e., Active sessions and FFR to low frequencies). Specifically, when comparing time-domain SNR values from the total portion of the FFR (Active Low Baseline vs. Post: *d* = −0.054, CI [−0.050, 0.366]), small size effects were obtained, as well as confidence intervals including 0, thus suggesting the lack of effect at the population level. The same results were obtained for mean spectral amplitude at F0 (Active Low Baseline vs. Post: *d* = 0.033, CI [−0.53, 0.44]) as well as in spectral SNR (Active Low Baseline vs. Post: *d* = −0.155, CI [−0.61, 0.25]). Additional comparisons in other portions of the FFR (i.e., constant or transient), as well as with FFR to high-frequency stimuli and sham sessions, showed similar results and are reported in Supplementary Table 1.

**FIGURE 3:**
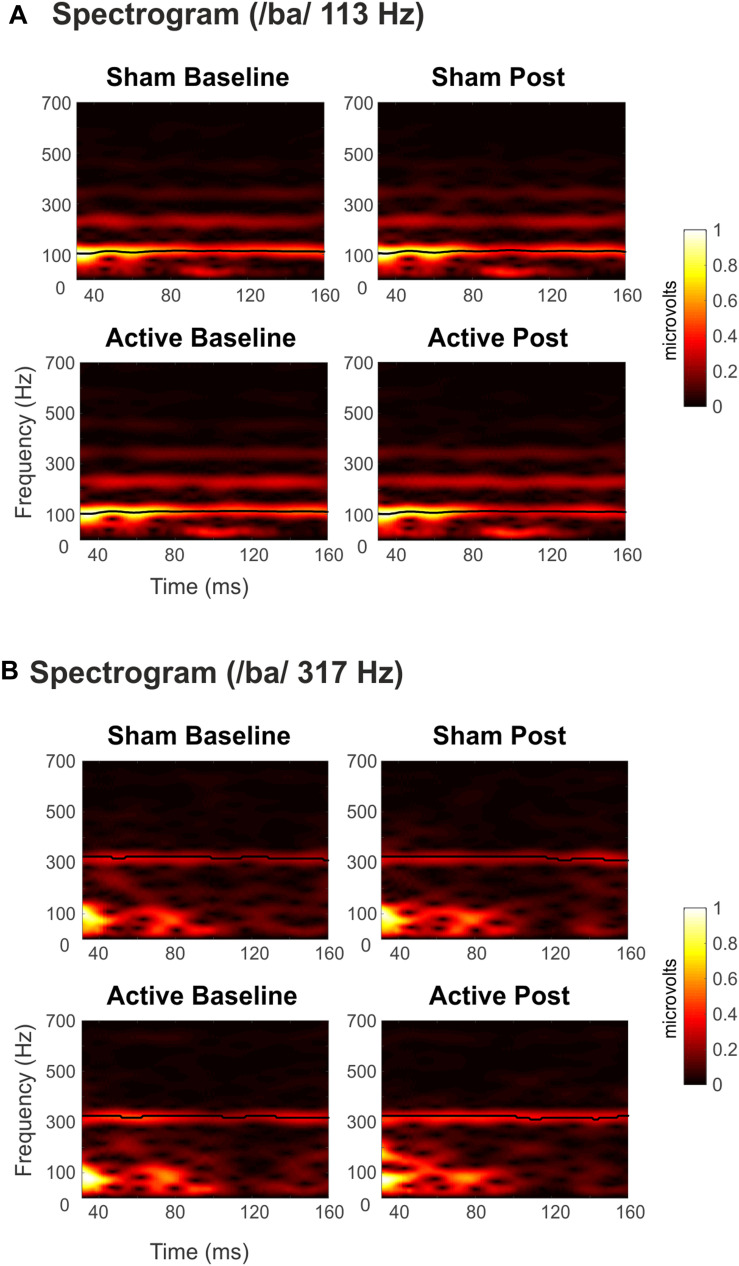
Spectrograms of the grand-averaged FFRs elicited to syllable /ba/ with **(A)** Low (113 Hz) and **(B)** High (317 Hz) F0. Top panels for Sham sessions and bottom ones for Active sessions. Baseline measurements to the left, Post cTBS measurements to the right. Darkest to lighter colors indicate spectral amplitude (μV) from lower to higher values, as a function of time and frequency. The black line shows time points with maximum amplitudes.

**FIGURE 4:**
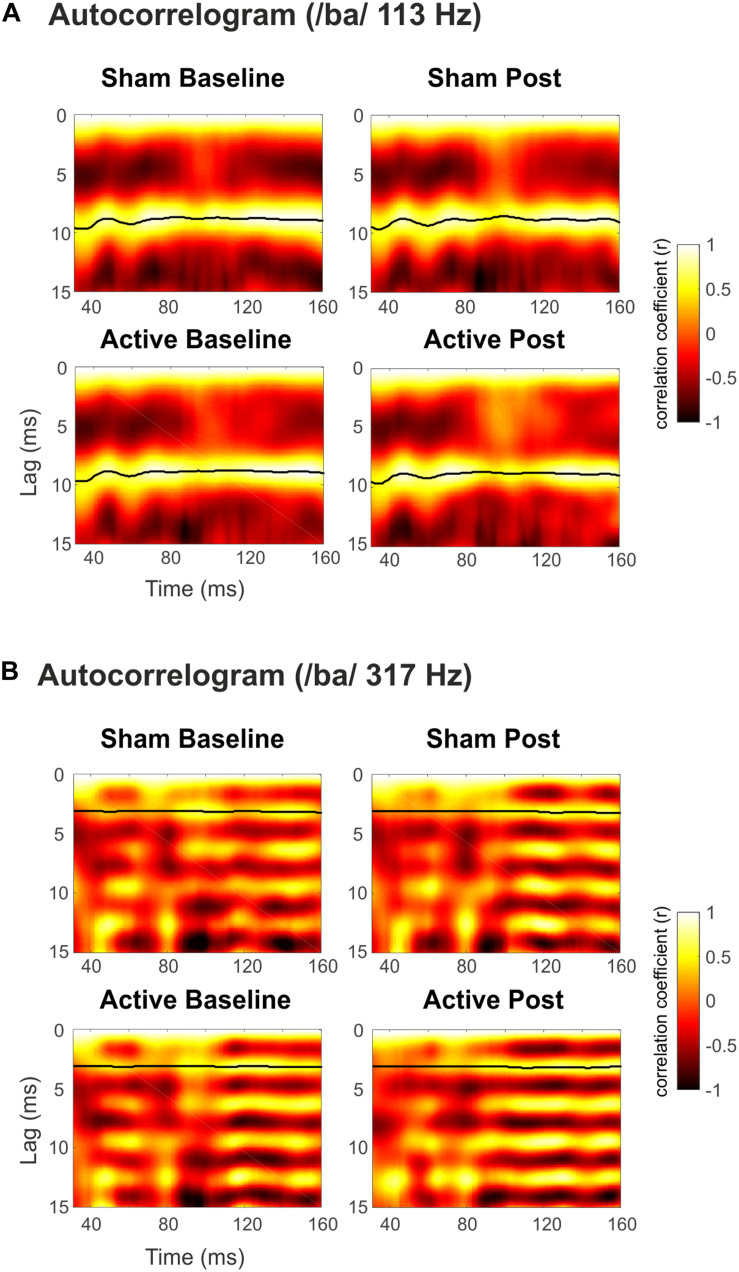
Autocorrelogram of the grand-averaged FFRs elicited to syllable /ba/ with **(A)** Low (113 Hz) and **(B)** High (317 Hz) F0. Top panels for Sham sessions and bottom ones for Active sessions. Baseline measurements to the left, Post cTBS measurements to the right. Darker to lighter colors indicate autocorrelation values from –1 to 1 (Pearson’s *r*), as a function of time and lag. The black line shows time points with maximum autocorrelation values.

Grand-average spectrograms were also computed for illustrative purposes (Figures 3A,B), where maximum amplitudes can be observed at frequencies corresponding to syllables F0 along the duration of the stimuli. Observable harmonics are also present in the FFR to low frequencies. Moreover, autocorrelogram plots (Figures 4A,B), for both low- and high-frequency stimuli, show the FFR phase-locking to the stimulus F0. Statistical comparisons for pitch strength measures revealed a main effect of Frequency [*F*_(__1_,_19__)_ = 57.411, *p* < 0.001, $\eta_{\text{p}}^{2}$ = 0.751], thus showing that the robustness of the response’s phase-locking to the syllable F0 contour (Jeng et al., 2013) was higher for FFR to low-frequency stimuli. The same effect on the Frequency factor was found for pitch error, reflecting higher-pitch encoding accuracy for low-frequency FFR [*F*_(__1_,_19__)_ = 76.600, *p* < 0.001, $\eta_{\text{p}}^{2}$ = 0.801] and for maximum stimulus-to-response cross-correlation [*F*_(__1_,_19__)_ = 36.006, *p* < 0.001, $\eta_{\text{p}}^{2}$ = 0.655]. However, none of these measures yielded significant effects among Session or Measurement factors, or their interaction (Table 2), with no effects attributable to cTBS. Again, statistical comparisons with Cohen’s *d* for the main comparison of interest confirmed the lack of significant differences between Baseline and Post measurements in any of these FFR measures (Active Low Baseline vs. Post: pitch strength, *d* = −0.044, CI [−0.56, 0.40]; pitch error, *d* = 0.119, CI [−0.10, 0.36], max stimulus-to-response cross correlation, *d* = 0.056, CI [−0.20, 0.26]). Additional comparisons can be found in Supplementary Table 1.

**TABLE 2:** Results of repeated-measures ANOVA on FFR measures: first maximum stimulus-to-response cross-correlation (Maxcorr; Pearson’s *r*), Pitch strength (Pearson’s *r*), and Pitch error (Hz).

FFR statistics	*F*	*p*	*$\eta_{\text{p}}^{2}$*
**Maxcorr**			
Session	0.008	0.928	0.000
Measurement	2.173	0.157	0.103
Ses*Meas	0.370	0.550	0.019
Ses*Meas*Fre	0.701	0.413	0.036
**Pitch strength**			
Session	1.964	0.177	0.094
Measurement	0.042	0.839	0.002
Ses*Meas	1.763	0.200	0.085
Ses*Meas*Fre	0.060	0.809	0.003
**Pitch error**			
Session	0.842	0.370	0.042
Measurement	0.379	0.545	0.020
Ses*Meas	0.004	0.951	0.000
Ses*Meas*Fre	0.000	0.998	0.000

Session factor includes Sham and Active levels, Measurement factor includes Baseline and Post levels, and Frequency factor includes Low- and High-frequency levels. Session and Measurement factors, the interaction between them (Ses*Meas), and a triple interaction between Session, Measurement, and Frequency (Ses*Meas*Fre) are reported. For each factor and their interaction, F- and p-values are presented (degrees of freedom: 1,19) along with effect sizes ($\eta_{p}^{2}$). p-values in the table are non-corrected for multiple comparisons.

Overall, from all these different approaches to the FFR data, we obtained no differences between Baseline and Post recordings in neither the Sham session nor the Active session, regardless of the stimulus fundamental frequency. Despite not being reported in the tables, neither the interaction between Frequency and Measurement nor Frequency and Session factors were significant for any of the FFR measures. Furthermore, the hypothesized triple interaction between Session, Measurement, and Frequency was not found (see Tables 1, 2), in any of the FFR measures studied.

Importantly, as a possible confounding factor in our results, we considered the distance between the stimulation target (right primary auditory cortex) and the nearest point on the surface of the head, where the center of the coil was placed. Such distance was calculated in every participant as the amount of 1-mm MRI slices, moving in the sagittal plane (*x*- coordinates), from the stimulation target to the last MRI slice with head tissue in the corresponding *y*- and *z*- coordinates (mean distance 30.45 mm, standard deviation 1.82 mm, range from 28 to 34 mm). However, adding these participant individual distances as a covariable for our ANOVA analysis did not produce significant results either. In fact, Pearson’s *r* correlations performed between coil-to-target distance and the different amplitude measures obtained for FFR, ABR, and cortical potentials yielded small correlation coefficient values in all cases (*r* < 0.5).

### ABR and Cortical Potentials

**FIGURE 5:**
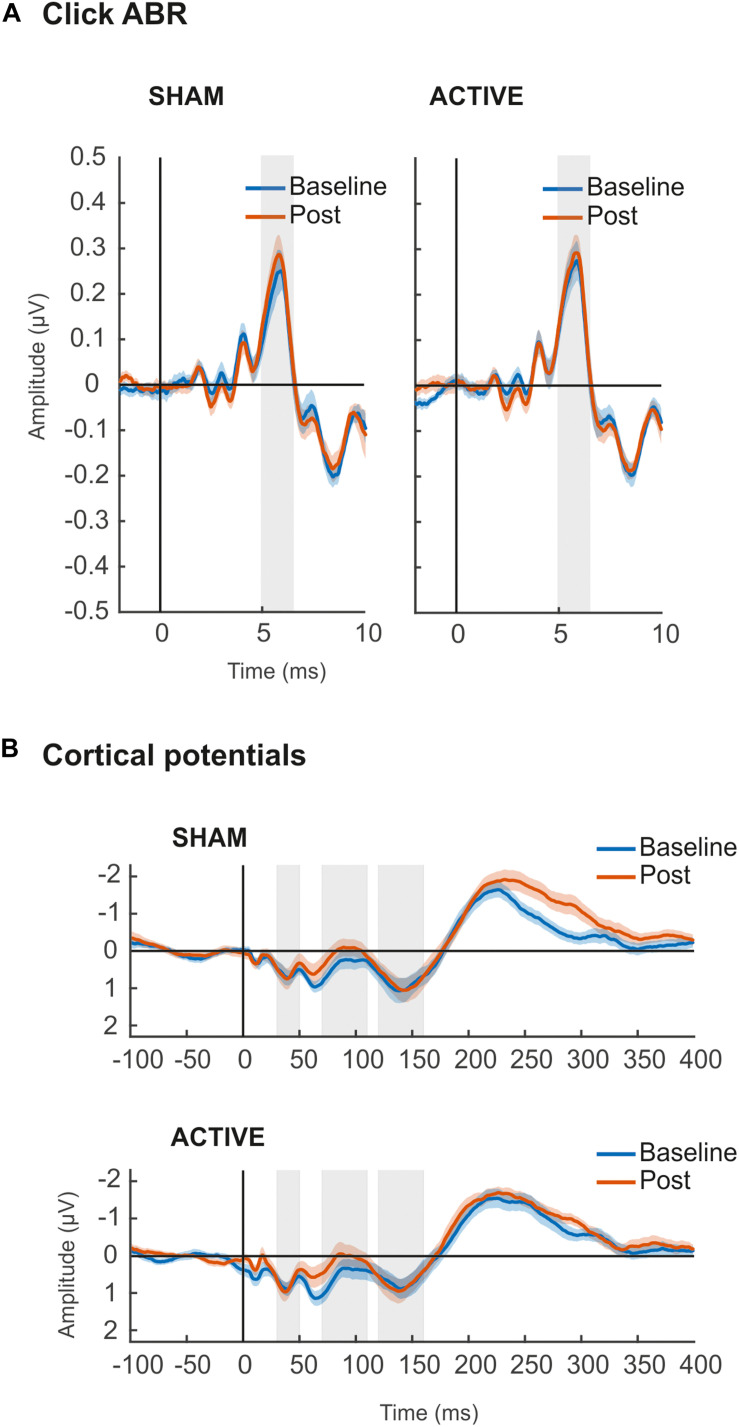
**(A)** Grand-averaged ABR waveforms elicited by auditory click stimulus in Sham (left) and Active (right) sessions. Shaded areas (5–6.5 ms) represent time windows of wave V, for where mean amplitude values (μV) were taken for statistical comparisons. **(B)** Grand-averaged waveforms of cortical evoked responses elicited by up-chirp stimulus in Sham (top) and Active (bottom) sessions. Shaded areas represent time windows of P50 (30–50 ms), N1 (70–110 ms), and P2 (120–160 ms) components, for where mean amplitude values (μV) were taken for statistical comparisons. For all figures, in blue, baseline recordings before cTBS pulse; in orange, Post cTBS recordings.

The amplitude of ABR wave V (Figure 5A) was overall larger [*F*_(__1_,_19__)_ = 4.539, *p* = 0.046, $\eta_{\text{p}}^{2}$ = 0.193] in the Post measurements (Table 3), although no interaction with the session factor was found, thus suggesting no effect of cTBS applied to the right primary auditory cortex in subcortical auditory evoked potentials (AEPs). Cohen’s *d* analyses confirmed such negative results (Active Baseline vs. Post, *d* = −0.133, CI [−0.30, 0.008]; Sham Baseline vs. Post, *d* = −0.235, CI [−0.43, 0.016]), with small size effects and confidence intervals including 0 value.

**TABLE 3:** Results of repeated-measures ANOVA on ABR wave V (5–6.5 ms) and cortical components P50 (30–50 ms), N1 (70–110 ms), and P2 (120–160 ms) amplitude values (μV).

ABR/cortical	*F*	*p*	*$\eta_{\text{p}}^{2}$*
**Wave V ABR**			
Session	2.162	0.158	0.102
Measurement	4.539	**0.046**	0.193
Ses*Meas	0.685	0.418	0.035
**P50**			
Session	0.433	0.519	0.022
Measurement	1.009	0.328	0.050
Ses*Meas	0.045	0.834	0.002
**N1**			
Session	0.093	0.764	0.005
Measurement	1.878	0.187	0.090
Ses*Meas	0.463	0.505	0.024
**P2**			
Session	4.074	0.058	0.177
Measurement	1.172	0.292	0.058
Ses*Meas	0.000	0.955	0.000

Session factor includes Sham and Active levels, and Measurement factor includes Baseline and Post levels. Session and Measurement factors, as well as the interaction between them (Ses*Meas), are reported. For each factor and their interaction, F- and p-values are presented (degrees of freedom: 1,19), along with effect sizes ($\eta_{p}^{2}$). P-values below 0.05 are highlighted. *p-values in the table are non-corrected for multiple comparisons*.

Cortical potentials (Figure 5B) were not affected by the cTBS pulse either. Specifically, mean amplitudes of the cortical potentials analyzed, P50, N1, and P2, were not significantly different across measurements or sessions, and no significant interaction between these two factors was found in ANOVA (Table 3). Cortical potentials were a crucial indicator in our study to prove the effect of cTBS on the auditory cortex, yet we found no significant results. Results from Cohen’s *d* and confidence intervals also pointed toward the lack of significant differences between Baseline and Post mean amplitude values in either Active (P50, *d* = −0.134, CI [−0.59, 0.49]; N1, *d* = −0.317, CI [−0.04, 0.80]; P2, *d* = −0.160, CI [−0.74, 0.34]) or Sham (P50, *d* = −0.184, CI [−0.55, 0.15]; N1, *d* = −0.144, CI [−0.37, 0.51]; P2, *d* = −0.151, CI [−0.45, 0.18]) sessions.

### Additional Control: Experiment With Cortical Potentials

Since we did not observe any effect of the cTBS on cortical evoked potentials, we argued that one possible reason for that was the fact that the cortical potentials recording blocks were the last ones acquired after the cTBS administration (e.g., 21 min) and therefore the potential inhibitory effects may have vanished by that time (see Wischnewski and Schutter, 2015; Chung et al., 2016). To control for such possibility, an additional experiment was conducted a few months after the completion of the original one in a subsample of 11 participants from the original study, who voluntarily took part in it. In this control experiment, we used the exact same parameters as described in the methods section, with the exception that only a single block of cortical potentials before and after the cTBS pulse (Baseline and Post) was recorded. In such block, four recordings of cortical potentials with 200 artifact-free sweeps each were acquired, in both Sham and Active sessions. The acquisition time for each of these four recordings was 1′ 40″. Among these four recordings, two were using the same up-chirp stimuli from the original study, and the other two, interspersed between those, were using a pure tone of 880 Hz and 100 ms duration. Therefore, the cortical potential block followed the sequence: Chirp – Pure Tone – Chirp – Pure Tone, with the starting type of stimuli counterbalanced across subjects. The rationale behind the use of additional recordings of cortical potentials in this new experiment was to assess whether the hypothetical cTBS effects would be present at cortical potentials recorded immediately after the cTBS pulse but fade away in the successive recording, although such effect was not observed. Moreover, this time, we aimed to test cTBS effects both with the stimuli used in the original study and with a pure tone, since this second stimulus elicited a larger N1 response.

**FIGURE 6:**
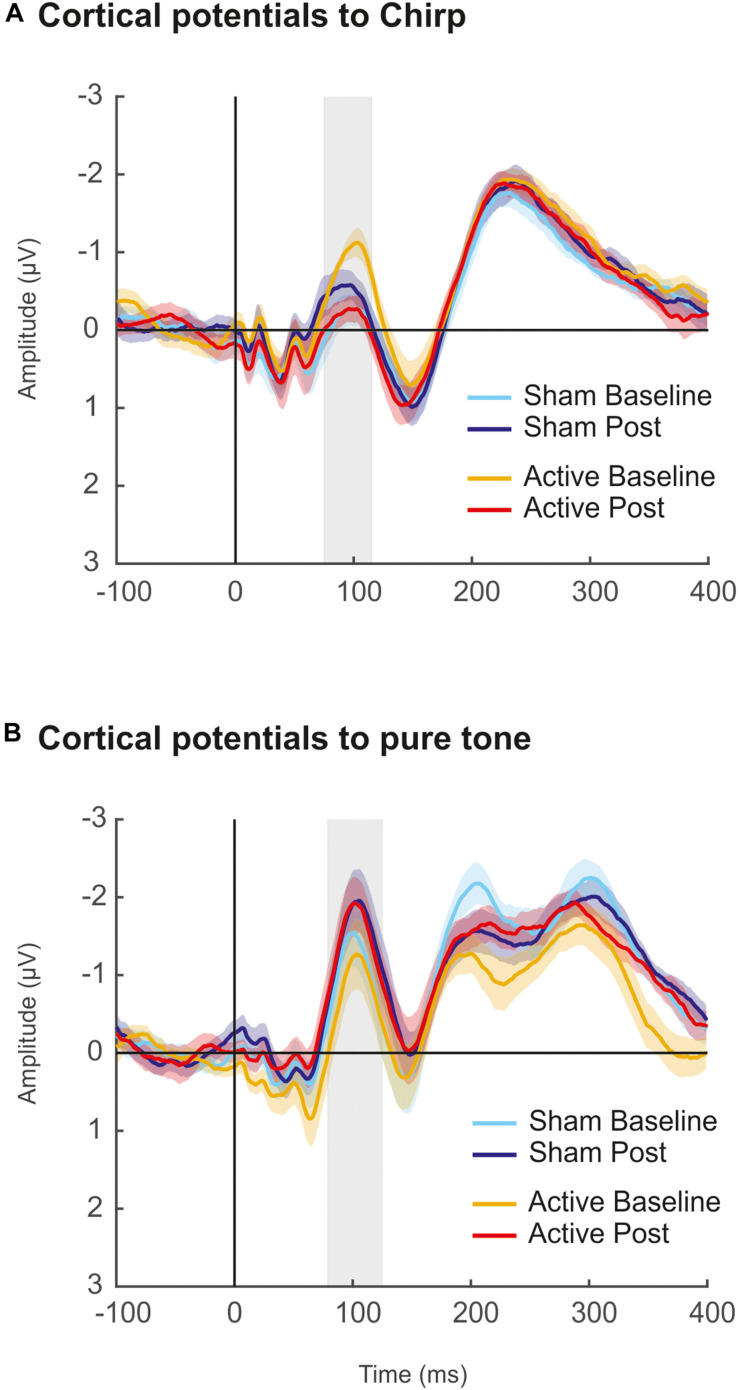
Results of additional control experiment. **(A)** Grand-averaged waveforms of cortical evoked responses elicited by up-chirp stimulus in Sham (cool colors) and Active (warm colors) sessions. The shaded area (75–115 ms) represent the time window of the N1 component, for where mean amplitude values (μV) were taken for statistical comparisons. **(B)** Grand-averaged waveforms of cortical evoked responses elicited by pure tone stimulus in Sham (cool colors) and Active (warm colors) sessions. The shaded area (75–125 ms) represents the time window of the N1 component, for where mean amplitude values (μV) were taken for statistical comparisons. For all figures, lighter colors refer to baseline recordings before cTBS pulse; darker ones correspond to Post cTBS recordings.

Results from the additional experiment are shown in Figure 6. Cortical potentials were obtained by averaging trials from the two recordings within each Session (Active, Sham), Measurement (Baseline, Post), and stimulus type (Pure Tone and Chirp stimulus), and mean amplitudes at the N1 peak (75–115 ms for evoked responses to chirp, 75–125 ms for evoked responses to pure tone; defined based on the grand-average waveforms) were retrieved for statistical analyses. Two-way repeated-measures ANOVA revealed no significant effects of Session factor [*F*_(__1_,_10__)_ = 0.493, *p* = 0.499, $\eta_{\text{p}}^{2}$ = 0.047] and Measurement factor [*F*_(__1_,_10__)_ = 0.805, *p* = 0.391, $\eta_{\text{p}}^{2}$ = 0.074] for N1 elicited by the chirp stimulus. A significant interaction between Session and Measurement factors was found [*F*_(__1_,_10__)_ = 10.56, *p* = 0.009, $\eta_{\text{p}}^{2}$ = 0.514], reflecting the different direction of the N1 amplitude changes after cTBS, with an increase in the sham session from Baseline to Post measurements and a decrease in the active one from Baseline to Post. For N1 elicited by the pure tone stimulus, a main effect of Measurement [*F*_(__1_,_10__)_ = 7.119, *p* = 0.024, $\eta_{\text{p}}^{2}$ = 0.416] was found, thus revealing that, overall, N1 amplitude changed between Baseline and Post measurements, but regardless of the session. No effects were found for Session factor [*F*_(__1_,_10__)_ = 1.487, *p* = 0.251, $\eta_{\text{p}}^{2}$ = 0.129] and Session*Measurement interaction [*F*_(__1_,_10__)_ = 0.841, *p* = 0.381, $\eta_{\text{p}}^{2}$ = 0.078]. Further statistical testing with Cohens’s *d* and confidence intervals revealed moderate to strong size effects when comparing Baseline to Post mean N1 amplitudes elicited by chirp stimulus, but such effects were found both in Active (*d* = −1.0658, CI [−1.84, −0.02]) and Sham sessions (*d* = 0.533, CI [0.09, 0.96]). With pure tone stimulus, a moderate size effect was found for Baseline vs. Post comparison in Active session (*d* = 0.481, CI [0.008, 1.10]), with confidence intervals excluding the 0 value, but similar results were found in the Sham session (*d* = 0.309, CI [0.02, 0.75]). Moreover, no differences were found between N1 amplitudes of the two Post measurements (Sham vs. Active: *d* = −0.10, CI [−0.57, 0.18]).

In summary, the results of the additional control experiment ruled out the attribution of the lack of effects in cortical potentials to the time delay between the administration of the cTBS pulse and the recording of these responses, and they further confirmed the lack of effects of cTBS over the right primary auditory cortex on cortical potentials. Indeed, the fact alone that FFR measures were not affected in any of the stimulation frequencies could have driven the conclusion that the scalp-recorded FFR had no cortical contribution, as the transient inactivation of the right primary auditory cortex did not affect FFR measurements to any of the stimulation frequencies. However, given the results in cortical potentials, the lack of effects on the FFR measures cannot be attributed to the lack of cortical contribution to this evoked potential, provided the neural generators of both signals are in the same location within the auditory cortex. Furthermore, as performed for FFR recordings, adding participants’ individual target-to-surface distance as a covariable for all ANOVA analyses of ABR, cortical potentials, or additional experiment recordings did not reveal any significant effect attributable to cTBS.

Yet, to further confirm the statistical results obtained with standard tests in our study, we computed the Bayes factors for *t*-tests for the main statistical comparisons that yielded non-significant results, which allowed to better differentiate whether these were due to insensitive data or in favor of the null hypothesis (Rouder et al., 2009; Morey and Rouder, 2011). Using a default Scale *r* factor of 0.707, all Bayes factor results were in favor of the null hypothesis. We included these results in Supplementary Table 2.

## Discussion

In the present study, we applied cTBS, an inhibitory rTMS pulse, in the right primary auditory cortex aiming to produce a transient inactivation in this region that would last for approximately 30 min (Chung et al., 2016). Within this time window, we recorded FFR, ABR, and cortical potentials and tested whether these would be affected by the hypothesized transient inactivation. Specifically, our hypothesis was that FFR recorded to Low (113 Hz) stimulus fundamental frequency, as compared to High (317 Hz), would be modulated by that inactivation, provided the auditory cortex contributes to the FFR signal at low- but not high-stimulus frequencies. Moreover, cortical but not ABR potentials would be modulated as well, as the former have proven cortical contributions. Clear AEP were obtained in all sessions and conditions according to the literature, thus revealing appropriate recording and analysis protocols. In spite of the robust and compelling AEP obtained at all levels recorded (FFR, ABR, and cortical potentials), however, our results suggest no effect of cTBS on the auditory cortex, as cortical potentials were not affected, therefore leaving results on the FFR uninterpretable. Several reasons behind this lack of effects are discussed, which yield several highlights regarding the use of cTBS protocols in the auditory cortex.

The first possible cause of our negative findings is that the cTBS pulse was largely ineffective in our target area of stimulation, the primary auditory cortex. Reviewing the efficacy of rTMS protocols in producing transient inhibitory effects at the neuronal level, on the one hand, we find studies on neuron-enriched primary cortical cultures (Grehl et al., 2015) revealing that TBS protocols increase intracellular calcium, which can modulate synaptic plasticity (Hulme et al., 2012), leading to long-term depression mechanisms. Moreover, TBS regulates the expression of genes related with dendritic growth, which is associated with morphological changes in neuronal projections (Grehl et al., 2015). On the other hand, in humans, cTBS has been shown effective to produce long-term depression-like effects in a targeted neural population (Huang et al., 2005) (for a review, see Chung et al., 2016). However, in these last studies evidence comes from the measurement of activity from the motor cortex.

Importantly, only a few studies have addressed whether cTBS produces measurable changes in auditory cortical regions. Among them, a study combining fMRI with TMS (Andoh and Zatorre, 2013) applied cTBS over the right HG to a group of healthy individuals. Their results showed an increase in the BOLD response in contralateral areas of the auditory cortex, instead of the expected decrease in the targeted area, which, in turn, was related with a faster response time in a melody-processing task. In another study targeting the same area with cTBS (Andoh et al., 2015), the authors found connectivity decreases in auditory and motor-related networks during resting state and concluded that studies using inhibitory TMS protocols should take into account network-level effects. Some other studies have investigated the effect of cTBS in auditory areas with clinical populations, specially tinnitus. For instance, using functional near-infrared spectroscopy in tinnitus patients, it was found that cTBS produced changes in sound-evoked brain oxygenation in the primary auditory cortex, with reversed patterns for active and placebo conditions, as well as different results for block and event-related designs (Schecklmann et al., 2014). Moreover, clinical effects of repetitive TMS protocols, including cTBS, over the primary auditory cortex have been measured on tinnitus, aiming to reduce the symptomatology (Barwood et al., 2013; Schecklmann et al., 2016; Sahlsten et al., 2017). From these studies, only one (Barwood et al., 2013), with four patients, found significant improvement in tinnitus when comparing active and placebo TMS conditions. In the other two studies, improvement of tinnitus scores was not superior in active than in placebo conditions, thus suggesting no clinically relevant effects. Still within the auditory cortex, evidence on the inhibitory effects of rTMS comes also from studies on schizophrenia patients, in which the transient inactivation of areas within this region (e.g., HG, temporoparietal cortex, language areas determined with fMRI) is intended to ameliorate auditory hallucinations. To this regard, some studies show benefits from rTMS in reducing auditory hallucinations (Hoffman et al., 2003), whereas others find no differences between active and placebo groups (McIntosh et al., 2004; Blumberger et al., 2012; Paillère-Martinot et al., 2017), and several meta-analyses show an overall small but present effect of rTMS on auditory hallucinations (Aleman et al., 2007; Freitas et al., 2009). It must be noted, however, that in these studies the TMS target is typically located in the left hemisphere, as auditory hallucinations are attributed to language areas. To that regard, we may have missed the effects by targeting the right hemisphere. In summary, despite cTBS long-lasting inhibitory effects described at cellular (Grehl et al., 2015) and cortical (Huang et al., 2005; Chung et al., 2016) levels, and despite the cTBS modulation of the BOLD response and connectivity patterns when administered over the right primary auditory cortex (Andoh and Zatorre, 2013; Andoh et al., 2015), no clear conclusions on the inhibitory effects of cTBS or rTMS over the right auditory cortex can be drawn from the literature. Future studies should further address this issue, perhaps including tasks to assess whether these inhibitory effects modulate behavioral measures of auditory processing, which we could not include in the present design due to the limited time window of cTBS inhibitory effects.

In comparison to effects on auditory cortical areas, repeated TMS protocols applied to other sensory areas of the cortex, including primary ones, have been proven to produce robust effects. For instance, rTMS impaired motion discrimination and accuracy when applied in the primary visual cortex and secondary areas (Thompson et al., 2016). In the somatosensory cortex, cTBS over primary somatosensory areas (S1) impaired tactile acuity (Rai et al., 2012), rTMS over S1 impaired the processing of contralateral visual stimuli of human body parts being touched only by human agents (Rossetti et al., 2012), and rTMS over S2 produced changes in BOLD response in the area and decreased the participant’s ratings of touch intensity [using a H8 deep TMS coil (Case et al., 2017)]. Notably, a crucial element when considering the effectiveness of any TMS protocol in cortical areas is how deep within the brain the stimulation target is. In addition to fMRI-measured and sensory processing effects observed in the studies described, several studies measuring rTMS effects on event-related potentials, as we do, have targeted areas of superficial cortex [e.g., prefrontal (Lowe et al., 2018; Sokhadze et al., 2018) or somatosensory (Poreisz et al., 2008) areas]. However, our target area of stimulation, the primary auditory cortex, includes a part of cortex buried within the temporal lobe, at the supratemporal plane [i.e., Heschl’s gyri (Abdul-Kareem and Sluming, 2008)]. Considering that our pulse intensities were determined as 80% of aMTH, following safety guidelines, the possibility exists that our TMS pulses were not reaching the target area, although it is also plausible that other more lateral regions of the superior temporal plane, still within the auditory cortex, were affected instead. The particular location of the primary auditory cortex, in fact, may help explain the overall more consistent findings in the literature on rTMS inhibitory effects on visual or somatosensory primary areas of the cortex, in comparison with auditory ones. Moreover, determining the intensity of the TMS pulse using the motor cortex (superficial cortex) as a reference, again following standard procedures, may undermine our success when trying to target an area of cortex with a greater separation from the coil than the motor cortex.

A different but related interpretation on the lack of effects observed in the present study would be that, despite cTBS producing a transient inactivation of the auditory cortex, the effects were not reflected in the AEPs recorded. To this regard, to the best of our knowledge, no previous study has addressed whether cTBS over the auditory cortex affects AEPs. There are, however, studies combining EEG and TMS over the auditory cortex using paired associative stimulation (PAS), that is, pairing external acoustic stimuli with TMS pulses applied to the corresponding cortical region where stimuli would be processed (Stefan et al., 2000). When performing PAS so that the TMS pulse occurs right before the incoming acoustic stimulus, for instance, long-term depression-like mechanisms can be generated, reducing synaptic connectivity. In these studies, several AEPs were modulated when performing PAS protocols over the auditory cortex, including N1–P2 complex (Schecklmann et al., 2011), auditory steady-state responses (Engel et al., 2017), or late AEPs (Markewitz et al., 2019). These studies demonstrate that, indeed, TMS over auditory cortical areas can modulate AEPs. Despite their findings, the kind of TMS protocols used in these designs, with almost simultaneous EEG recordings and TMS pulses, differs considerably from our protocol with cTBS, in which EEG recordings were separated in time from TMS administration. The possibility remains that cTBS, despite showing no effect on AEPs in our study, modulates non-phase-locked cortical activity. Given the findings on cTBS modulation of fMRI BOLD response (Andoh and Zatorre, 2013; Andoh et al., 2015), induced oscillatory activity in the cortex may indeed be affected by TMS. Unfortunately, the EEG acquisition equipment used in the present study did not allow for the retrieval of the EEG signal on single-trial basis, preventing us from performing any type of time–frequency analysis.

Compensatory mechanisms from the non-targeted areas contributing to the signal recorded could also potentially explain our negative results. Following international standards for the use of rTMS (Rossi et al., 2009), the administration of cTBS was restricted to only one hemisphere. In our case, we chose the right one as our primary goal was to assess FFR, as contribution from the right hemisphere to this evoked potential was found to be more prominent (Coffey et al., 2016; Hartmann and Weisz, 2019; Ross et al., 2020). However, the possibility remains that the left auditory cortex compensates for the transient inactivation of the right one. Indeed, contralateral activation of the auditory cortex (Andoh and Zatorre, 2013) and temporoparietal areas (Tracy et al., 2010) compensating right-sided administration of rTMS has been described in healthy individuals. This possibility applies as well to our cortical potentials, the control condition to demonstrate the effect of cTBS in the present study, as long-latency potentials such as N1 are known to have contributions from both auditory cortices (Näätänen and Picton, 1987; Recasens et al., 2012), as well as from frontal areas, such as premotor cortices, supplementary motor area, or anterior cingulate (Näätänen and Picton, 1987; Alcaini et al., 1994; Giard et al., 1994; Picton et al., 1995; Bender et al., 2006). These areas could in fact partially contribute to the amplitude of components. Therefore, a transient inactivation of a small area of the right auditory cortex may have been insufficient to affect the amplitude of the signal to a significant degree.

A further important element to consider when performing cTBS protocols is the effectiveness interindividual and intraindividual variability described for the pulse in motor areas (Vallence et al., 2015; Jannati et al., 2017), also including the direction (suppressive or facilitatory) of the effects (Hamada et al., 2012). Importantly, some studies performing rTMS protocols (Martin-Trias et al., 2018) and intermittent theta burst stimulation (López-Alonso et al., 2014) in the dorsolateral prefrontal cortex and motor cortex, respectively, report responsiveness values in approximately 40% of participants. Several factors have been described as influencing this variability in motor areas (Martin-Trias et al., 2018), including coil orientation discrepancies among studies (Talelli et al., 2007) and subject factors such as age (Todd et al., 2010), gender (Chaieb et al., 2008), genetics (Cheeran et al., 2008; Jannati et al., 2017), or relative levels of excitability in neuronal populations, affected by participants’ individual state [levels of fatigue, sleep or wakefulness, etc. (Silvanto et al., 2008; Hordacre et al., 2017)]. Considering that this variability has been described in areas of the motor cortex, we do not know to what extent it could be influencing results in a much less studied area such as the auditory cortex, and therefore, it constitutes a relevant factor to understand the lack of confirmation of our hypothesis.

## Conclusion

The present study addresses an important question in the field of auditory neuroscience, such as the neural origins of the FFR, and uses a novel and methodologically rigorous approach to answer it, alternative to EEG source-reconstruction techniques, by combining EEG and cTBS. No effects of cTBS were observed in FFR or cortical potentials, suggesting that the inactivation of an auditory sensory area with this protocol is ineffective. Nevertheless, this absence of effects is of particular relevance (The importance of no evidence n.d., 2019), as this is, to the best of our knowledge, the first attempt to record AEPs after a cTBS pulse in the primary auditory cortex. Moreover, possible reasons behind this lack of effects are discussed, which may be relevant to other studies using cTBS protocol in auditory cortical areas.

## Data Availability Statement

The datasets generated for this study are available on request to the corresponding author.

## Ethics Statement

The studies involving human participants were reviewed and approved by Bioethics Committee of the University of Barcelona. The patients/participants provided their written informed consent to participate in this study.

## Author Contributions

FL-C, DB-F, and CE designed the study. FL-C, PM-T, and NG-C performed the data acquisition. FL-C and TR-P carried out the data analysis. FL-C, DB-F, and CE wrote the manuscript. All authors revised and approved the final version of the manuscript.

## Conflict of Interest

The authors declare that the research was conducted in the absence of any commercial or financial relationships that could be construed as a potential conflict of interest. The reviewer GB declared a past supervisory role with one of the authors FL-C to the handling editor.
