# Supplementary material for: Effects of cTBS on the Frequency-Following Response and Other Auditory Evoked Potentials
Source: Front Hum Neurosci. 2020 Jul 8;14:250. doi: 10.3389/fnhum.2020.00250 (PMC7360924; doi:10.3389/fnhum.2020.00250)
Supplement: Supplementary file 1 [file Data_Sheet_1.PDF]

Supplementary materials for the article “Effects of cTBS on the Frequency-Following Response and other auditory evoked potentials”, by Fran López-Caballero, Pablo Martin-Trias, Teresa Ribas-Prats, Natàlia Gorina-Careta, David Bartrés-Faz and Carles Escera

Suppl. Table 1. All statistical comparisons performed over FFR measures using Cohen’s d and confidence intervals approach. In green, confidence intervals excluding 0.

**Measure**	snr_Tra_SHAM_LOW_PRE_VS_POST	snr_Tra_SHAM_HIGH_PRE_VS_POST
**Cohen d**	-0,399167089	-0,610720881
**CI 1**	-0,742792811	-0,897855366
**CI 2**	-0,062457349	-0,394212443

**Measure**	snr_Tra_ACTIVE_LOW_PRE_VS_POST	snr_Tra_ACTIVE_HIGH_PRE_VS_POST
**Cohen d**	-0,102697662	-0,491367407
**CI 1**	-0,462739042	-0,766238247
**CI 2**	0,234786915	-0,204793329

**Measure**	snr_Con_SHAM_LOW_PRE_VS_POST	snr_Con_SHAM_HIGH_PRE_VS_POST
**Cohen d**	-0,154861007	-0,105771925
**CI 1**	-0,447881494	-0,336393545
**CI 2**	0,277902515	0,096503934

**Measure**	snr_Con_ACTIVE_LOW_PRE_VS_POST	snr_Con_ACTIVE_HIGH_PRE_VS_POST
**Cohen d**	-0,022163401	-0,185620223
**CI 1**	-0,492423057	-0,562313188
**CI 2**	0,418258022	0,036919904

**Measure**	snr_tot_SHAM_LOW_PRE_VS_POST	snr_tot_SHAM_HIGH_PRE_VS_POST
**Cohen d**	-0,273318533	-0,429771967
**CI 1**	-0,606078294	-0,684849263
**CI 2**	0,12431973	-0,211102086

**Measure**	snr_tot_ACTIVE_LOW_PRE_VS_POST	snr_tot_ACTIVE_HIGH_PRE_VS_POST
**Cohen d**	-0,05437714	-0,314079175
**CI 1**	-0,505632931	-0,656877262
**CI 2**	0,366275042	-0,044203633

**Measure**	amp_Tra_SHAM_LOW_PRE_VS_POST	amp_Tra_SHAM_HIGH_PRE_VS_POST
**Cohen d**	-0,155095437	0,00233788
**CI 1**	-0,375126962	-0,371425684
**CI 2**	0,084583837	0,237561087

**Measure**	amp_Tra_ACTIVE_LOW_PRE_VS_POST	amp_Tra_ACTIVE_HIGH_PRE_VS_POST
**Cohen d**	-0,044270076	-0,10788563

**CI 1**	-0,348011386	-0,374900041
**CI 2**	0,258695613	0,064866273
**Measure**	amp_Con_SHAM_LOW_PRE_VS_POST	amp_Con_SHAM_HIGH_PRE_VS_POST
**Cohen d**	-0,013137175	-0,001507276
**CI 1**	-0,422713612	-0,125484473
**CI 2**	0,407948447	0,109522643
**Measure**	amp_Con_ACTIVE_LOW_PRE_VS_POST	amp_Con_ACTIVE_HIGH_PRE_VS_POST
**Cohen d**	0,056981459	-0,020278233
**CI 1**	-0,539448769	-0,3318611
**CI 2**	0,472465077	0,185903059
**Measure**	amp_tot_SHAM_LOW_PRE_VS_POST	amp_tot_SHAM_HIGH_PRE_VS_POST
**Cohen d**	-0,083026406	-0,013421627
**CI 1**	-0,449101835	-0,127014914
**CI 2**	0,341997434	0,121701056
**Measure**	amp_tot_ACTIVE_LOW_PRE_VS_POST	amp_tot_ACTIVE_HIGH_PRE_VS_POST
**Cohen d**	0,033161628	-0,010721878
**CI 1**	-0,530311904	-0,231775278
**CI 2**	0,448251284	0,127368281
**Measure**	snr_amp_Tra_SHAM_LOW_PRE_VS_POST	snr_amp_Tra_SHAM_HIGH_PRE_VS_POST
**Cohen d**	-0,499728602	0,146670272
**CI 1**	-1,029186403	-0,293625401
**CI 2**	-0,174627695	0,596136183
**Measure**	snr_amp_Tra_ACTIVE_LOW_PRE_VS_POST	snr_amp_Tra_ACTIVE_HIGH_PRE_VS_POST
**Cohen d**	-0,284799233	-0,130967017
**CI 1**	-0,630483858	-0,52354689
**CI 2**	0,005710431	0,350437157
**Measure**	snr_amp_Con_SHAM_LOW_PRE_VS_POST	snr_amp_Con_SHAM_HIGH_PRE_VS_POST
**Cohen d**	-0,095249898	-0,024195792
**CI 1**	-0,421779635	-0,289634728
**CI 2**	0,284757647	0,247242394
**Measure**	snr_amp_Con_ACTIVE_LOW_PRE_VS_POST	snr_amp_Con_ACTIVE_HIGH_PRE_VS_POST
**Cohen d**	-0,037318794	0,362726576
**CI 1**	-0,555091891	-0,048084585
**CI 2**	0,372039801	0,821194355
**Measure**	snr_amp_Con_ACTIVE_LOW_PRE_VS_POST	snr_amp_Con_ACTIVE_HIGH_PRE_VS_POST
**Cohen d**	-0,037318794	0,362726576
**CI 1**	-0,555091891	-0,048084585

**CI 2**	0,372039801	0,821194355
**Measure**	snr_amp_tot_SHAM_LOW_PRE_VS_POST	snr_amp_tot_SHAM_HIGH_PRE_VS_POST
**Cohen d**	-0,067374129	0,019401104
**CI 1**	-0,419345621	-0,321844371
**CI 2**	0,31798107	0,287492076
**Measure**	snr_amp_tot_ACTIVE_LOW_PRE_VS_POST	snr_amp_tot_ACTIVE_HIGH_PRE_VS_POST
**Cohen d**	-0,155568586	0,315563043
**CI 1**	-0,612700842	-0,074030273
**CI 2**	0,252893974	0,739572116
**Measure**	maxcorr_SHAM_LOW_PRE_VS_POST	maxcorr_SHAM_HIGH_PRE_VS_POST
**Cohen d**	-0,063711189	0,214364145
**CI 1**	-0,337065173	0,025542447
**CI 2**	0,152275352	0,470076938
**Measure**	maxcorr_ACTIVE_LOW_PRE_VS_POST	maxcorr_ACTIVE_HIGH_PRE_VS_POST
**Cohen d**	0,056936524	0,218852252
**CI 1**	-0,205742963	0,007878677
**CI 2**	0,263745484	0,547160926
**Measure**	lag_SHAM_LOW_PRE_VS_POST	lag_SHAM_HIGH_PRE_VS_POST
**Cohen d**	-0,206625614	0,024224404
**CI 1**	-0,496028404	-0,473655841
**CI 2**	0,015779113	0,459579135
**Measure**	lag_ACTIVE_LOW_PRE_VS_POST	lag_ACTIVE_HIGH_PRE_VS_POST
**Cohen d**	-0,194577094	0,275985583
**CI 1**	-0,897938617	-0,381139285
**CI 2**	0,137744864	0,938960909
**Measure**	Pitch_strength_SHAM_LOW_PRE_VS_POST	Pitch_strength_SHAM_HIGH_PRE_VS_POST
**Cohen d**	-0,285816623	0,033534023
**CI 1**	-0,766880349	-0,223262428
**CI 2**	0,220671526	0,262319181
**Measure**	Pitch_strength_ACTIVE_LOW_PRE_VS_POST	Pitch_strength_ACTIVE_HIGH_PRE_VS_POST
**Cohen d**	-0,044025535	0,207090778
**CI 1**	-0,561015711	-0,076977144
**CI 2**	0,400236443	0,702789833
**Measure**	Pitch_Error_SHAM_LOW_PRE_VS_POST	Pitch_Error_SHAM_HIGH_PRE_VS_POST
**Cohen d**	0,135917252	0,000672874
**CI 1**	-0,14258452	-0,344738527
**CI 2**	0,582499524	0,192600719

Measure	Pitch_Error_ACTIVE_LOW_PRE_VS_POST	Pitch_Error_ACTIVE_HIGH_PRE_VS_POST
**Cohen d**	0,119532095	-0,005707051
**CI 1**	-0,103243344	-0,234874242
**CI 2**	0,364789553	0,258170075

Suppl. Table 2. Results of Bayes Factors for t-test for the main statistical comparisons that yielded non-significant results in our study (in all cases, they support the null hypothesis)

	Scaled JZS Bayes Factor	Scaled-Information Bayes Factor
**FFR SNR time domain-total Active Low**	4.202013	3.233414
**FFR F0 amplitude-total Active Low**	4.268901	3.287816
**FFR SNR spectral F0-total Active Low:**	3.738302	2.857945
**Maxcorr Active Low: Baseline vs Post**	3.818093	2.922336
**Pitch strength Active Low: Baseline vs Post**	4.239722	3.264077
**Pitch error Active Low: Baseline vs Post**	2.47751	1.853981
**Wave V ABR Active: Baseline vs Post**	1.247251	0.9054836
**P50 Active: Baseline vs Post**	3.83035	2.932236
**N1 Active: Baseline vs Post**	1.609795	1.180977
**P2 Active: Baseline vs Post**	3.715752	2.839764
